# The immune landscape during the tumorigenesis of cervical cancer

**DOI:** 10.1002/cam4.3833

**Published:** 2021-03-10

**Authors:** Yiying Wang, Mengdi He, Guodong Zhang, Kankan Cao, Moran Yang, Hongwei Zhang, Haiou Liu

**Affiliations:** ^1^ Obstetrics and Gynecology Hospital Fudan University Shanghai China; ^2^ Shanghai Key Laboratory of Female Reproductive Endocrine Related Diseases Obstetrics and Gynecology Hospital Fudan University Shanghai China

**Keywords:** cervical carcinogenesis, immune checkpoint, immune evasion, immunotherapy, tumor environment

## Abstract

**Objective:**

Deciphering the determinants of the intralesional immune reaction in cervical carcinogenesis may be conducive to improving the understanding of the disease and then improve outcomes.

**Methods:**

Public gene‐expression data and full clinical annotation were searched in Gene Expression Omnibus in the joint analysis of the array‐based four eligible cohorts. The infiltrating estimation was quantified using microenvironment cell populations‐counter algorithm and absolute‐mode CIBERSORT and verified by flow cytometry analysis. An unsupervised classification on immune genes strongly associated with progression, designated by linear mixed‐effects regression. We determined immune response and signaling features of the different developmental stages and immune phenotypes by functional annotation and systematically correlated the expression of immune checkpoints with cell‐infiltrating characteristics.

**Results:**

We identified the lesion‐intrinsic immunosuppression mechanism was triggered at precancerous stages, such as genome instability and mutation, aerobic glycolysis, activation of proto‐oncogene pathways and so forth. Predominant innate and adoptive cells were increasing from normalcy to cancer (B cell, total T cell, regulatory T cells [Tregs], monocytes, neutrophils, and M2‐like macrophages) together with the decrease of CD4^+^ T cell and CD8^+^ T cell through the development of cervical cancer. Immune escape initiated on the expression of immunosuppressive molecules from high‐grade squamous intraepithelial lesions (HSIL) and culminated in squamous cell carcinoma (SCC). Of note, the expression of immune checkpoints was escalated in the immune‐hot and immune‐warm phenotype largely encompassed by HSIL and SCC under the stress of both activated and suppressive immune responses.

**Conclusions:**

Immune surveillance is unleashing from low‐grade squamous intraepithelial lesions onwards and immune‐suppression mechanisms are triggered in HSIL. Thorough knowledge of the immune changing pattern during cervical tumorigenesis contributes to finding the potential therapeutic targets to susceptive patients towards immune checkpoints inhibitors.

## Graphical abstract

Immune surveillance is ignited from low‐grade squamous intraepithelial lesions onwards in cervical disease. High‐grade squamous intraepithelial lesions (HSIL) become the kick‐starter of immune escape during cerivcal carcinogenesis. Immunoactive HSIL may benefit from immune checkpoint inhibitors.

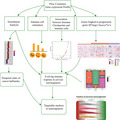

## 1 INTRODUCTION

Despite being largely preventable through early vaccination and screening strategies, cervical cancer still ranks fourth for both incidence and mortality among females.^1^ It often takes several years to develop abnormal stages into malignant cancers. Thus, the range of consecutive pre‐invasive stages preceding squamous cell carcinoma (SCC) in the cervix makes it an accessible model for studying the early evolution of cancer critically.

One of the best‐known theories that tumor and immune cells interact in a dynamic equilibrium shaping the progression of the disease. Immune escape of immune surveillance is a hallmark by all types of cancer.^2^ In this process, the features that protect abnormal cells from attack by cytotoxic immune cells^3^, ^4^ or promote the infiltrates of immunosuppressive cells^4^, ^5^ are positively selected. Additionally, one of the mechanisms for immune evasion—the activation of immune checkpoints—has been therapeutically explored in preclinical trials of cervical cancer.^6^ Although studies have demonstrated relatively high programmed cell death protein 1 (PD‐1)/programmed cell death protein ligand‐1 expression on cervical tumors, there is an insufficient study showing the expression of immunosuppressive molecular in preinvasive stages.^7^, ^8^ Thus, the discovery of features acquired by abnormal cells in response to the immune cells surrounding them may shed light on new strategies to treat the disease.

Gene Expression Omnibus (GEO) has provided abundant data to identify the distinguishing molecular traits of cervical carcinogenesis in detail. The research demonstrated considerable metabolic shifts, consistent cell proliferation, and DNA repair during cervical carcinogenesis.^9^ Although several recent studies have explored some features of the cervical diseases associated with specific characteristics of the immune infiltrates,^4^, ^5^, ^10^ a comprehensive progressive landscape using computational methods of the interactions between the tumor and the immune cells is still lacking. Figuring the underlying mechanisms of immune escape out may set the stage for therapies to prevent and intercept the development of cancer or personalized immunotherapy in cervical carcinogenesis.^11^

Here, we first aimed to determine to what extent the developmental stages shape the immune infiltrate of cervical diseases. To this end, we estimated the immune infiltration pattern across the spectrum of cervical diseases, compared to normal tissues. Our results showed the pathological stages do not explain all the variability of the immune infiltration. Therefore, we set out to identify intrinsic immune features in cervical diseases (immune phenotypes). These immune phenotypes represent distinct scenarios of immune infiltration, and then, we reasoned that a fraction of high‐grade squamous intraepithelial lesions (HSIL) showed the propensity of immune evasion. As a result, we revealed cervical diseases develop molecular traits and evasive mechanisms while progressing. Our findings provide a landscape of the interactions between abnormally and immune cells and have clear implications for immunotherapies in the preinvasive stages.

## 2 MATERIALS AND METHODS

### 2.1 Patients and specimens

Tissue specimens from 26 patients with cervical diseases and normal tissues (Table S1) who were under the biopsy of the colposcope, conization, and surgery between 2018 and 2020 were obtained from the Gynecology and Obstetrics Hospital of Fudan University. The study was approved by the institutional ethics board in Obstetrics and Gynecology Hospital of Fudan University, and written consent form was finished.

### 2.2 Preparation of single cells from cervical tissues

Fresh tissues were washed 3 times with cold phosphate‐buffered saline (PBS) before being minced into small pieces. The specimens were collected in RPMI 1640 medium containing 1 mg/ml of collagenase II. Dissociated cell suspensions were further incubated for 1.5 h at 37°C under continuous rotation. The cell suspensions were then filtered through a 100‐µm cell strainer (BD), washed once with PBS, and resuspended in cell staining buffer.

### 2.3 Flow cytometry

Single‐cell suspensions were stained with a panel of fluorochrome‐tagged antibodies (Table S2). Samples were incubated with Live/Dead Fixable Dead Cell Staining Kit (ThermoFisher) and human BD Fc Block (BD Biosciences), then stained with the indicated monoclonal antibodies (mAbs) for 30 min at 4°C in dark. When necessary, before staining with antibodies against intracellular proteins or transcription factors, cells were pretreated with Fixation/Permeabilization Solution Kit or Transcription Factor Fixation/Permeabilization buffer (BD Biosciences), respectively, according to the manufacturer's instructions. For sample acquisition, a Beckman Coulter cytoflex flow cytometer with FACS CytExpert software was used (Beckman Coulter), and FlowJo software (Tree Star) was used for analyses.

### 2.4 Gene expression profiles of cervical disease

We download the publicly available cervical disease expression datasets from the GEO, uploaded up to 28 March 2020. Cohorts with ≥20 samples were selected.

In total, four eligible cervical disease cohorts were derived from GPL570 (GSE5787, GSE63514, GSE75132, and GSE27678). Analysis in the present study was confined to samples measured via the Affymetrix Human Genome U133 Plus 2.0 Array. We downloaded the raw “CEL” files and adopted a robust multiarray averaging method with the affy^12^ and simpleaffy^13^ packages to perform background adjustment and quantile normalization. We performed a quality check, platform‐specific normalization, and combined them by gcrma package. Duplicate samples were removed from the meta‐cohort. Batch effects from nonbiological technical biases were corrected using the “ComBat” algorithm of sva package. The baseline information of all eligible cervical disease datasets was summarized in Table S3.

### 2.5 Differentially expressed genes between different pathological stages and distinct immune phenotypes

Differentially expressed genes (DEGs) were performed by the R package “limma”^14^ using the standard comparison model. *p* values were adjusted for multiple testing using an embedded Benjamini–Hochberg (BH) procedure in the package. The significance criteria for determining DEGs were set as the adjusted *p* value <0.05 and |log_2_FC| ≥ 1.

### 2.6 Enrichment analysis of gene set and pathway

Separate regulating patterns of genes were respectively enriched in default gene sets by the portal web site of Metascape,^15^ within the range of gene ontology (GO), Kyoto Encyclopedia of Genes and Genomes, Hallmark database and Reactome, etc. default pathways. We then loaded the individual matrix on Cytoscape software version 3.7.1.^16^ One analysis per subset was performed. ClueGO Application was used to determine pathway enrichment in each network. Public datasets only from “Experimental evidence” of GO—ImmuneSystemProcess—GOA (updated date: 4 September 2018) were used. The Go Term Fusion option was selected. Only pathways with a BH adjusted *p* value below 0.05 were kept.

Gene set enrichment analysis (GSEA) relied on all the DEGs to provide more specific information on the Hallmark pathway, using the R package “clusterProfiler”^17^ with the number of permutation (nPerm) set to 1000 permutations. Hallmark gene sets were downloaded from the MSigDB database.^18^ Visualization methods in this research were implemented in the R package “DOSE.”^19^ Significant results are designated by *q* value <0.05.

Ultimately, single‐sample GSEA (ssGSEA) was used to specifically characterize the immune status of immunophenotyping through gene signature sets from established published studies by R package GSVA.^20^

### 2.7 Estimates of infiltrating immune cells

To estimate the absolute proportions of eight immune cells and two stromal cells in the cervical disease samples, we used the R microenvironment cell populations‐counter (MCP‐counter)^21^ package to quantify the cell composition of bulk tissues from gene expression and recorded them. Also, the absolute‐mode CIBERSORT algorithm based on the LM22 gene signature was performed. Gene expression profiles were prepared using standard annotation files, and data were uploaded to the CIBERSORT “absolute mode” (http://cibersort.stanford.edu/), with the algorithm run using 1000 permutations. From all the samples analyzed, we have selected 21/9/44/59 Normal/LSIL/HSIL/SCC samples respectively which met the requirements of CIBERSORT *p* value <0.05. Lastly, xCell infers cell abundance in an available website via enrichment of gene signatures from 64 cell types.^22^

### 2.8 Identification of linear immune gene‐expression changes and molecular phenotypes

Immune gene‐expression alterations during carcinogenesis were identified using a linear model with mixed effects. For each gene, a linear mixed‐effects model was fitted over the *n* = 225 samples using the R function lmer in the package lme4.^23^ Each gene was modeled as a function of the developmental stage (factor variable), adjusting for technical differences between sample batches and biological differences among the four histopathological tissue classes as the fixed effect. Random patient effects accommodated a possible correlation between multiple tissues from the same patient. Analysis of variance (ANOVA) tests compared the association of a gene and developmental stage to a null model. The false discovery rate (FDR) was calculated for each ANOVA *p* value using the Benjamini–Hochberg method, which was used in multiple hypothesis testing to correct for multiple comparisons. Six hundred sixty‐one genes significantly associated with developmental stages were determined by FDR <0.00001. Then, the set of 22 altered immune‐genes generated by filtering the mentioned significant sets with the 770 genes related to the human PanCancer Immune Profiling panel.^24^ Unsupervised hierarchical clustering of these genes was then used to compare the four different developmental stages (based on Euclidean distance and complete linkage).

### 2.9 Statistical analysis

Mann–Whitney *U* test was utilized for comparisons of two groups. Kruskal–Wallis test was used to conduct different comparisons of three or more groups, and *p* values were adjusted for multiple testing using an embedded BH procedure. Correlation analyses were computed using Spearman's rank correlation coefficient test. Statistical analyses were performed with SPSS Statistics 21.0, R (version 3.6.3) and R Bioconductor packages. All statistical *p* values were two‐sided, with *p* < 0.05 as statistically significant.

## 3 RESULT

### 3.1 Temporal order of cancer hallmarks during carcinogenesis

**FIGURE 1:**
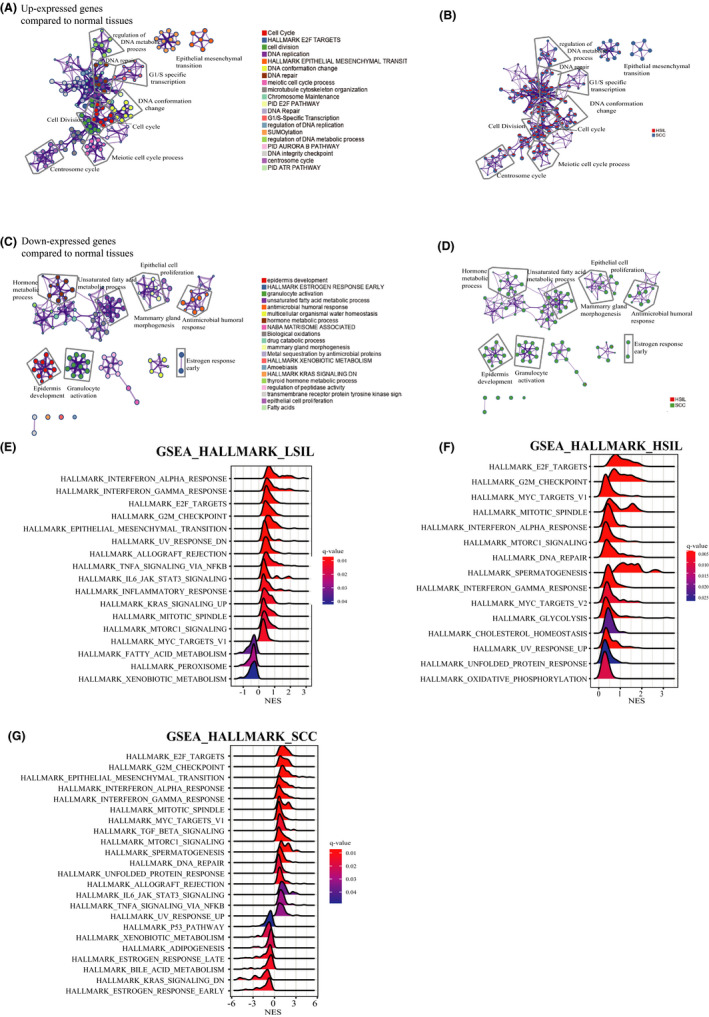
Temporal order of cancer hallmarks during carcinogenesis. (A,B) Network showing the top 30 terms in over‐representation analysis (ORA) of Metascape default pathways between transformed and normal stages, including up‐expressed gene list in (A) and color of pies based on the identities of the gene lists in (B). (C,D) Network showing the top 30 terms in ORA of Metascape default pathways between transformed and normal stages, including downregulated gene list in (C) and color of pies based on the identities of the gene lists in (D). (E–G) Gene sets enrichment analysis (GSEA) plots showing enrichment of the Hallmark pathway in different developmental stages. (E) in low‐grade squamous intraepithelial lesions (LSIL), (F) in high‐grade squamous intraepithelial lesions (HSIL), and (G) in squamous cell carcinoma (SCC)

Carcinogenesis has been viewed as the process of acquiring advantageous biological capabilities by abnormal cells. We identified the DEGs from premalignant and malignant tissues to normal tissues and then examined them for analysis of pathway enrichment (Figure 1A–D). We discovered only upregulated genes enriched to significant results from both HSIL and SCC stages, namely without the participation of the low‐grade squamous intraepithelial lesions (LSIL). The cellular proliferation process and DNA repair pathway were commonly upregulated in both HSIL and SCC stages. Especially, the epithelial‐mesenchymal transition only upenriched in SCC compared with HSIL (Figure 1A,B; Figure S1A,B). Moving on to functionally inhibitive pathways, both immunosuppressive and metabolic pathways were discerned, to name but a few concerned, antimicrobial humoral response, granulocyte activation, estrogen response early, hormone metabolic process, and unsaturated fatty acid metabolic process (Figure 1C,D). It was reasonable to detect the epithelial cell proliferation and development were inhibited in SCC.

To verify what have found specifically, MSigDB Hallmark pathway enrichment was analyzed with all DEGs by GSEA (Figure 1E–G). Across the disease spectrum, all DEGs from different stages showed consistent activation in immune response (interferon‐α and interferon‐γ responses), cell proliferation including E2F targets, G2M checkpoint and MYC target, MITOTIC spindle, and MTORC1 signaling pathway (Figure 1E–G). The inhibitive pathways were gathering in metabolism at both LSIL and SCC stages. To put it concisely, xenobiotic metabolism was inhibited in both LSIL and SCC, and the same as fatty acid metabolism in LSIL and estrogen response together with bile acid metabolism in SCC (Figure 1E,G). As for the DNA damage, UV response was downregulated in LSIL, then UV response was activated together with DNA repair in HSIL and SCC. Strikingly, DEGs in HSIL were merely involved in the activation of pathways, such as cell proliferation, DNA damage (DNA repair and UV response up), immune response, metabolism (glycolysis, cholesterol homeostasis, oxidative phosphorylation), unfolded protein response pathway, and spermatogenesis (Figure 1F). Moreover, epithelial‐mesenchymal transition predominated in both LSIL and SCC stages except for HSIL. In line with previous studies, TGF‐β signaling, estrogen response early, and p53 pathway were activated in SCC.^9^ Epithelial‐mesenchymal transition and the Janus kinase (JAK)/STAT3 pathway initiated in LSIL and boomed in SCC (Figure 1E,G). Additionally, the activation of the JAK/STAT3 pathway driven by IL‐6 was not identical to the previous prediction that strong STAT3 activation is representative in cervical high‐grade lesions and then withdraw in cervical cancer within tumor nests.^3^, ^25^

### 3.2 Changes of local immune cells and immune‐modulatory genes contribute to the immunosuppressive microenvironment during the tumorigenesis

**FIGURE 2:**
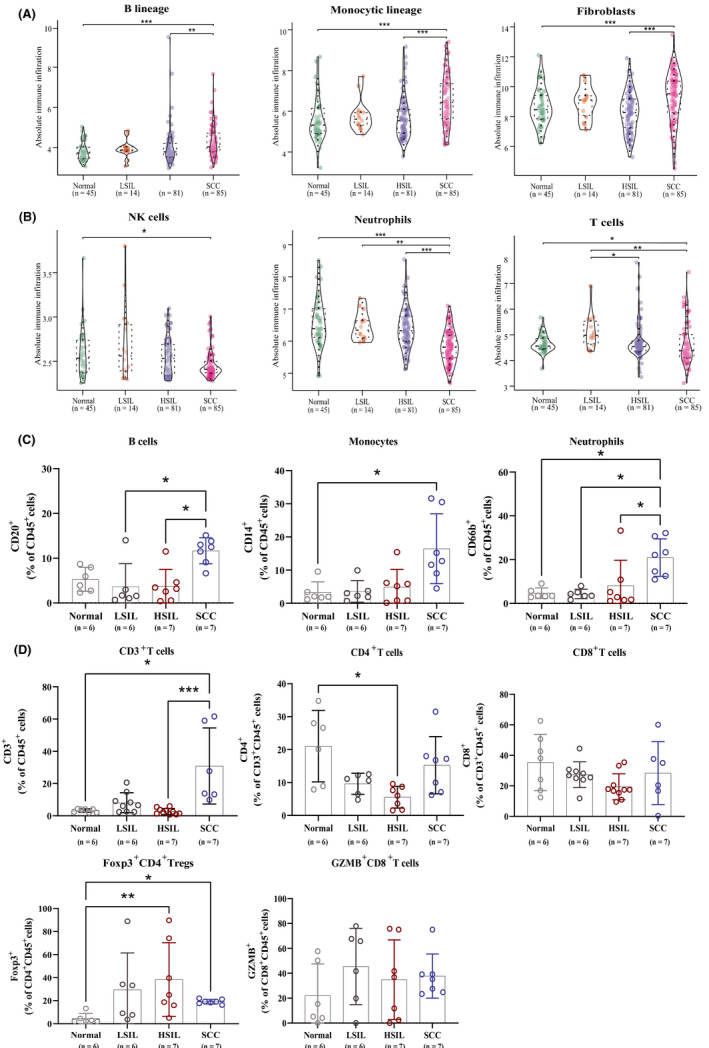
Changes in the composition of infiltrating immune cells during the tumorigenesis of cervical cancer. (A,B) Box plots showing differences in the abundance of T cells, B cells, monocytes, neutrophils, and macrophages by the estimation of microenvironment cell populations‐counter in cervical tissues during carcinogenesis. (C,D) Distribution of percentage of immune cells (CD20^+^ B cell, CD14^+^ monocytes, CD66b^+^ neutrophils, CD3^+^ T, CD4^+^ T, CD8^+^ T, Foxp3^+^ CD4^+^ Tregs, and GZMB^+^ CD8^+^ T) in normal cervical tissues and different stage of cervical carcinogenesis. *p < 0.05, **p < 0.01, and ***p < 0.001. HSIL, high‐grade squamous intraepithelial lesions; LSIL, low‐grade squamous intraepithelial lesions; SCC, squamous cell carcinoma

After a comparison of three methods for estimation of immune infiltrates (MCP‐counter, CIBERSORT and xCell) (Figure S3A,B), the enrichment approach (MCP‐counter) (Figure S3C) and deconvoluting method (CIBERSORT) (Figure S3D) are the optimal choice for our study to evaluate the immune infiltrates, following the results of flow cytometry gating on Figure S2. Typical results showed B lineages, monocyte lineages, and fibroblasts increased from normal stages to late developmental stages and specifically both significantly increased from HSIL to SCC (Figure 2A,C). Neutrophils were discerned significantly decreased in SCC and a slight decrease showed from normal tissues to SCC in NK cells (Figure 2B), whereas neutrophils were increasing from normalcy to cancer (Figure 2C). T cells showed the decreasing trend from normalcy and LSIL to HSIL with higher infiltration in normalcy, while bioinformatically T cell infiltrated lowest in cancer contradictive with experimental results (Figure 2B,D). As reported, CD4^+^ T and CD8^+^ T cells infiltrated higher in normalcy and cancer, lower in LSIL and HSIL stages (Figure 2D).^26^ The infiltration number of regulatory T cells (Tregs), GZMB^+^ cytotoxic T cells increased from normalcy to HSIL and fell from HSIL to cancer (Figure 2D). Of note, neutrophils and NK cells significantly higher than other stages (Figure 2C), which was the opposite to bioinformatical result showing the neutrophils was the significantly lowest in cancer (Figure 2B). Furthermore, macrophages congregated highest in normalcy and significantly lowest in HSIL compared with normal tissues, while M2‐like macrophages rose from normalcy to successive stages along with the significant increase from the normal cervix to LSIL (Figure S3E). Surprisingly, the changing pattern of the absolute immune score was identical to B lineage, monocytic lineage and fibroblasts (Figure 2A; Figure S3D).

**FIGURE 3:**
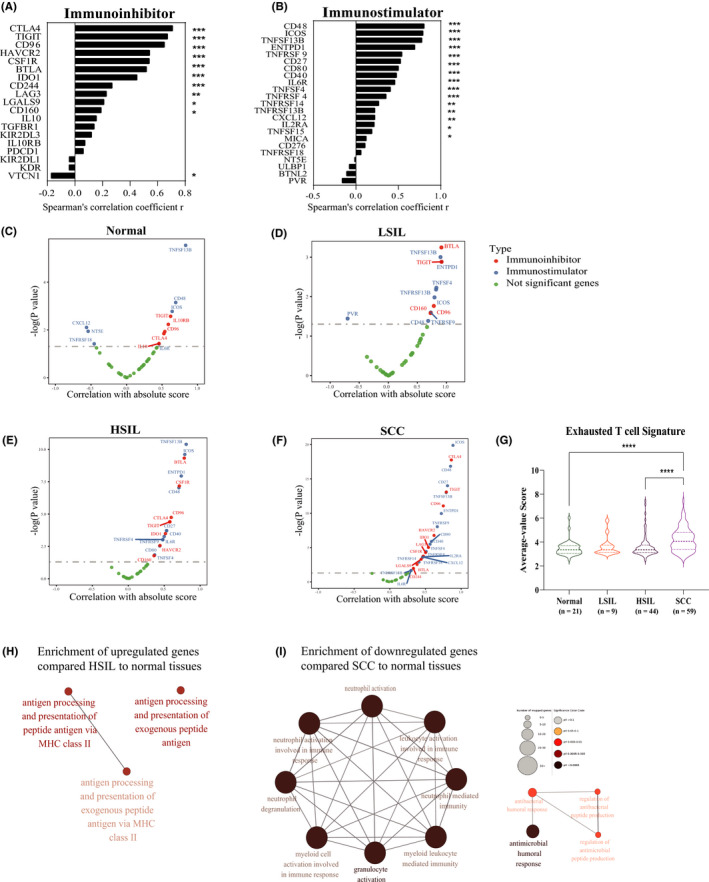
Immune evasion before tumor invasion in early cervical squamous carcinogenesis. (A) Spearman's correlation between absolute score and immunoinhibitive genes expression across the progression of normal to advanced stages. (B) Spearman's correlation between absolute score and immunostimulatory genes expression across the progression of normal to advanced stages. (C–F) A positive correlation between expression was observed for both immune‐suppressive and immune‐stimulatory genes and absolute score from the individual transformed stage to normal tissues. (G) Violin plots showing differences in exhausted T cell score among four steps during cervical carcinogenesis. (H,I) Network showing all enrichment terms in ORA of the immune‐related pathways between transformed and normal stages. Terms of pathways (along the perimeter) showing the immune‐related pathways (along the perimeter) most significantly overrepresented (FDR <0.05) in stages of cervical disease progression. Color represented the adjusted *p* value and size shows the number of mapped genes. Kruskal–Wallis tests. *p < 0.05, **p < 0.01, ***p < 0.001, and ****p < 0.0001. HSIL, high‐grade squamous intraepithelial lesions; LSIL, low‐grade squamous intraepithelial lesions; SCC, squamous cell carcinoma

Accounting for the heterogeneity of immune infiltration in different patients, it was an urge that closer examination of immunomodulatory gene expression correlating with the immune absolute score. Tables depicted the correlation coefficients between immune‐modulatory genes and the corresponding absolute score among all pathological stages (Figure 3A,B). What stood out in the tables was the patients afflicted with the cervical disease showed positive correlation significantly, such as famous immune checkpoint genes (*CTLA4*, *TIGIT*, *HAVCR2*, *LAG3*, etc.) and costimulatory genes like *ICOS*, *CD80*, *CD40*, and so forth (Figure 3A,B).

Furthermore, the expression pattern of immune‐modulatory genes in separately pathological stages intrigued us. In normal tissues, several expression values of immunomodulatory genes showed a significant correlation with the immune infiltrating score (*TIGIT*, *IL10RB*, *CD96*, and *CTLA4*), and even some immunostimulatory genes were negatively linked (*CXCL12*, *NT5E*, and *TNFRSF18*) (Figure 3C). Despite a slight increase in immune score, more immunostimulatory genes manifested a stronger association with immune infiltration than immune inhibitors in LSIL like *OX40* antigen (also known as *TNFRSF4* [or *CD134*]), *TNFRSF9* (also known as *4*‐*1BB* ligand receptor or *CD137*), *TNFRSF13B*, *ENTPD1* (also known as *CD39*), and *CD48* (also known as B‐lymphocyte activation marker [BLAST‐1] or signaling lymphocytic activation molecule 2 [SLAMF2]) (Figure 3D). De facto, we identified the correlation coefficients were significantly higher and also outnumbered in both HSIL and the succeeding stage (Figure 3E,F), even though there was a slight decrease from LSIL to HSIL in immune‐infiltration levels and marginal growth at the followed stage (Figure 3C,D). As well as suppressive molecules such as *CSF1R*, *CTLA4*, *IDO1*, and *HAVCR2*, immunostimulatory molecules such as *ICOS*, *CD27*, *CD40*, *CD80*, and *IL6R* showed an increased correlation in HSIL and, to a greater extent, at the invasive stage (Figure 3E,F). Each of the markers showed an increase in the correlation with immune infiltration in SCC compared to normal tissue, which was significant for *CTLA4*, *HAVCR2*, *IDO1*, and *LAG3* (*p* < 0.05) but not for *PDCD1* (*p* = 0.19) and *TIGIT* (*p* = 0.79) (Figure 3F). Taken together, immune escape occurred before tumor invasion, as proven by the progressively higher numbers and values of significant correlation coefficients in immunosuppressive genes from HSIL onwards.

Aim to figure out the specific shifts of immune status in the malignant transformation, we then calculated the exhausted T cell score with average expression values within the genes in signature^27^ and performed the overrepresentation analysis of the DEGs in the immune signatures collection as well. We noticed exhausted T cell score started rocketing from HSIL and reached the peak in SCC (Figure 3G), which also supported the former conclusion that immune escape occurred before malignancy. Turning to the latter result, only we acquire the results from the following two parts. One of which, few immune functions were specifically modulated for HISL—only among upregulated genes (*n* = 3 functions) (for instance, antigen processing and presentation of peptide antigen via MHC class II) (Figure 3H). The other in SCC, plenty of immune functions were uniquely down enriched (Figure 3I), suggesting granulocyte activation symbolized innate immunity and the antimicrobial humoral response was predominantly suppressed in cancerous patients. No matter the changing pattern of neutrophils was rising in experiments or shrinking bioinformatically (Figure 2B,C), together with the downregulation of granulocyte activation (Figure 3I), while increasing monocytic lineage from HSIL to SCC accompanied with activation of antigen processing and presentation in HSIL (Figures 2I and 3I).

### 3.3 Immune phenotype reveals characteristic features in cervical disease progression

**FIGURE 4:**
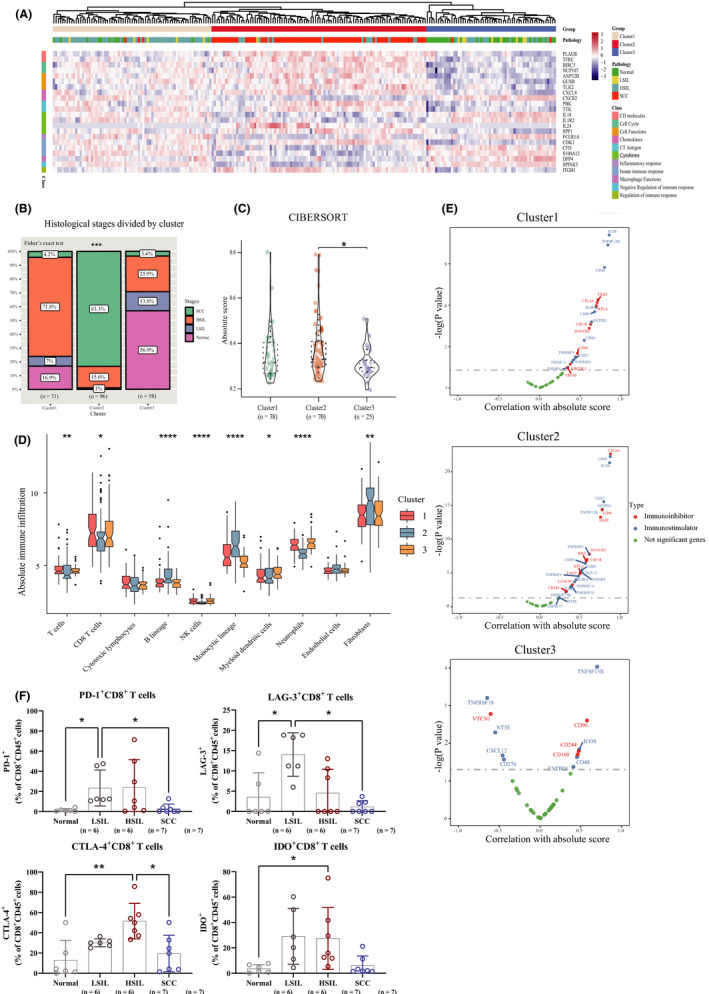
FIIntegration of immune signatures across the progression of cervical disease. (A) Unsupervised hierarchical clustering of cervical disease meta‐cohort revealed three immune subtypes, termed as the immune gene clusters 1–3, respectively. (B) Stacked bar chart showing component differences of histological stages among the three immune phenotypes. Fisher's exact *T* test. (C) Violin plot showing differences in the absolute score of immune cells among three gene clusters. (D) Box plots showing differences in the abundance of immune cells by the estimation of microenvironment cell populations‐counter among three gene clusters. (E) Spearman's correlation between absolute score and observed expression for immunostimulatory and immunosuppressive interleukins among three gene clusters. Kruskal–Wallis test. (F) Distribution of percentage of immune checkpoint molecules (PD‐1, LAG‐3, CTLA‐4, IDO) on CD8^+^ T cells in normal cervical tissues and different stages of cervical carcinogenesis. *p < 0.05, **p < 0.01, and ***p < 0.001, and ****p < 0.0001. HSIL, high‐grade squamous intraepithelial lesions; LSIL, low‐grade squamous intraepithelial lesions; SCC, squamous cell carcinoma

Unsupervised hierarchical clustering of tumorigenesis‐and‐immunology‐related genes, strongly associated with progression, produced three subtypes (Figure 4A) with distinct immune characteristics. We observed the immune‐hot module (“cluster 2”) with the highest expression value among the 22 tumorigenesis immunity genes, the lowest expression as the immune‐cold subtype (“cluster 3”), the last subtype (“cluster 1”) owing intermediate expression value as immune‐warm. Surprisingly, the immune‐hot and immune‐warm group was mainly composed of both HSIL and SCC tissues (Figure 4B). In general, the immune‐hot subtype ranked first and the immune‐cold phenotype came at the bottom, with the immune‐warm subtype second to last in overall immune infiltrates (Figure 4C). We discerned the peak infiltrating level of B lineage, monocytic lineage, and fibroblasts that emerged in immunoreactive phenotype among the three subtypes (Figure 4D). Meanwhile, T cells, CD8 T cells, NK cells, myeloid dendritic cells, and neutrophils showed the lowest infiltration in the immunoreactive subtype. Referring to the functional analysis in immune‐related pathways, we discovered the immune‐hot and immune‐warm phenotype was characterized by immunosuppression and pro‐inflammation (Figure 4E). To name but a few, exhausted T cells was the highest expression in cluster 2 (Figure S4A). TGF‐β and coinhibition on antigen‐presenting cells (APCs) played an important role in both immune‐hot and immune‐warm phenotype (Figure S4B), annotating the immunosuppressive was happening in precancerous stages accompanying with the anti‐tumoral responses, such as the IFN‐γ‐related response, antigen processing and presentation, and BCR signaling pathway (Figure S4C). Furthermore, immune modulators showed the most significantly activated in the immune‐hot subtype, including suppressive genes such as *CTLA4*, *CD96*, *TIGIT*, *HAVCR2*, *TGFBR1*, *IL10*, *PDCD1*, *IDO1*, and immunostimulatory genes like *ICOS*, *CD48*, *CD27*, *ENTPD1*, *TNFSF4*, *TNFRSF9*, *NT5E*, and so forth as well (Figure 4E). Meanwhile, the immune‐warm phenotype just followed the steps below the immune‐hot subtype and showed the relative association with both immunoactivity and immunosuppression, overlapping with *ICOS*, *TNFSF13B*, and *CD48* as costimulators and *CTLA*‐*4*, *TIGIT*, and *HAVCR2* as coinhibitors among the top‐three‐strong correlation between checkpoint expression and tumor‐infiltrating cells (Figure 4E). In summary, the immune‐hot and immune‐warm subtype manifested the strongest immunosuppressive activity accompanied by the activation of immune responses in the meantime.

As mentioned earlier, both immunoreactive and immunosuppressive cells contribute to the microenvironment in HSIL. After classification of the immune subtypes, we delved into the influence of immunosuppressive activity in heterogeneous contexture by focusing on the patients afflicted with HSIL. Inspection of our flow cytometry results among full‐range steps, we found the percentage of PD‐1^+^ CD8^+^ T cells, CTLA‐4^+^ CD8^+^ T cells, and IDO^+^ CD8^+^ T cells in CD3^+^ T cells grew significantly from normalcy to HSIL (Figure 4F); however, there was insignificant result in comparison of HSIL with normalcy of the percentage of TIM‐3^+^ CD8^+^ T cells and LAG‐3^+^ CD8^+^ T cells in CD3^+^ T cells (Figure 4F; Figure S4D). HSIL derived from the immune‐hot and immune‐warm subtype expressed higher immune modulators than immune‐cold subtypes, such as *TIGIT*, *IDO1*, *CTLA4*, and *HAVCR2* (Figure S4E). In summary, immunosuppressive responses predominated among the immune‐hot and immune‐warm clusters, and we suggested the anti‐CTLA4 and anti‐IDO as potential therapeutic markers towards both HSIL and SCC in immune‐hot and immune‐warm clusters, which was proved to be evidence‐based and promising at the transcriptional level.

## 4 DISCUSSION

**FIGURE 5:**
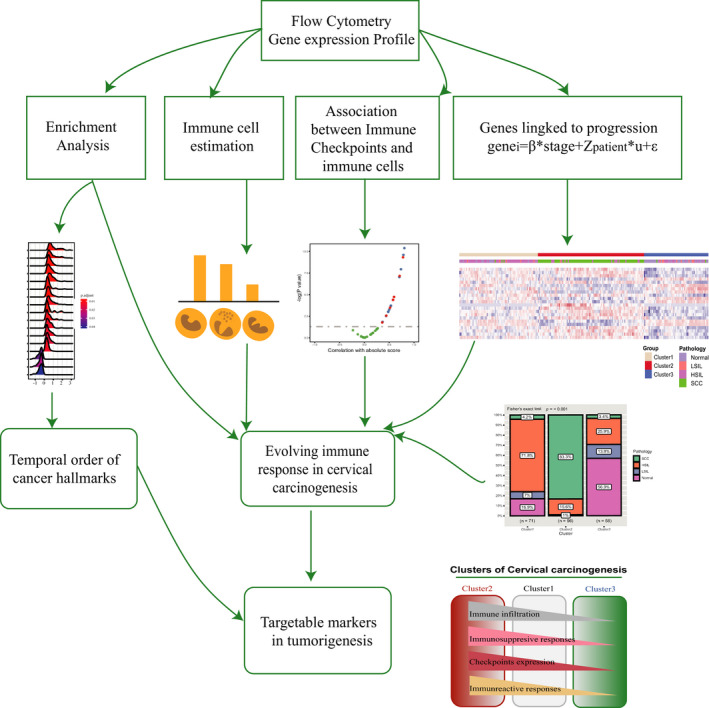
Model depicting the structured tumor‐immune microenvironment in patients afflicted with cervical disease. As demonstrated, we started by analyzing flow cytometry results and gene expression profiles to discover the temporal order of cancer hallmarks and evolving immune response in cervical carcinogenesis, then put forth the potential immunotherapeutic markers targeting susceptible patients. HSIL, high‐grade squamous intraepithelial lesions; LSIL, low‐grade squamous intraepithelial lesions; SCC, squamous cell carcinoma

Based on flow cytometry analysis and gene expression profiling of cervical disease, we uncovered both immune activation and immune suppression occur at precancerous stages of cancer development, which supports immune surveillance occurs at early stages, and HSIL is on the tipping point in the balance of immune activation and suppression. We checked the individual cohort data separately and ensured it was worth exploring the immune milieu of the tumorigenesis of the cervix from the whole progression perspective. Furthermore, the immune‐hot and immune‐warm subtypes, mainly accounting for both HSIL and SCC, show the propensity of increased inhibitive immune response together with escalating checkpoints expression annotating the HSIL and SCC patients from immune‐hot and immune‐warm clusters may benefit from immune checkpoint blockade (Figure 5).

Referring to significant events in cervical cancer development, early cervical lesions predominantly showed increased expression of genes with functions in DNA replication and cell division. This is consistent with the histological transition of the cervical epithelial from typically stratified toward gradually more convoluted epithelium in which most cells are actively dividing.^9^ It is important to reiterate that the epithelial‐mesenchymal transition is activated in both LSIL and cancer stage, which supports the dynamic historical changes accompanied with solely proliferation of epithelial cells in LSIL and annotates the metastatic process in cancer.^28^ Of note, glycolysis was activated in HSIL underlying even in the presence of oxygen, precancerous cells reprogram their glucose metabolism largely to glycolysis, similar with the hallmark of cancer.^2^

A range of immunosuppressive mechanisms occurs in the tumor microenvironment (TME), which hamper not only the natural host immune responses but the efficacy of cancer immunotherapies as well. Two types of immunosuppression mechanism exist in the TME—a tumor‐intrinsic and a local adaptive immune‐suppression.^29^ In SCC, the P53 inactivation may cause by the increased oncogenic human papillomavirus (HPV) protein E6 the binding with tumor suppressor protein (p53),^30^ resulting in uncontrolled cellular proliferation, DNA damage, and chromosomal instability.^31^ A dramatic decrease in estrogen receptor α expression throughout cervical cancer progression,^9^ as well as the down‐enriched early and late estrogen response discovered in our study. It is widely accepted that oncogenic KRAS is conductive to promote cell survival, proliferation, and cytokine secretion to acting on stromal cells to promote cancer malignancy by activating intracellular PI3K, MAPK, or RAL‐GEF pathways.^32^ KRAS signaling was upregulated in both LSIL and SCC to the normal stage, annotating the cell proliferation is thriving in stages. Meanwhile, we reasoned that the MYC targets and MTORC1 signaling is thriving through precancerous stages to malignancy compared with normalcy, resulting in drive tumorigenesis^33^ and decreased recruitment of T cells in the TME.^34^ Furthermore, stromal inflammation was ignited by HPV‐transformed cells activating the JAK/STAT3 signaling pathway in monocytes with the combination of the cytokine interleukin‐6 (IL‐6), macrophage colony‐stimulating factor and CCL2 from HSIL to SCC.^3^, ^25^ CCL2, a chemokine that promotes inflammation by the attraction of myelomonocytic infiltrates, also brings about pathological changes that promote progression towards neoplasia via the production of MMP‐9.^35^ Meanwhile, the gene *IL6R* expression showed a positive correlation with tumor‐infiltrating lymphocytes in HSIL, solidifying the activation of the JAK/STAT3 signaling pathway contributing to inflammation occurring in the very early stage and promoting the neoplasia.

Turning to dysregulation of the immune microenvironment, the protection of abnormal cells from attack by cytotoxic immune cells plays a pillar role in cancerous progression. We noticed the highest number of infiltrating CD3^+^ T cells in SCC among whole tumorigenesis steps and significantly higher than both HSIL and normal tissues (Figure 2D). Bioinformatically, we found there was contradictive with experimental results in the infiltration of CD3^+^ T cell in cancer which was the lowest in cancer (Figure 2B). We considered this was caused by the different portion of the stages of SCC in the experimental cohort and public‐data cohort, the former consisted of the early stages (I and II) patients, but the latter was heterogeneous. Elevated cytotoxic CD8^+^ T cells in the precancerous stage contribute to the regression of LSIL^10^ and become a good prognostic marker of cervical cancer.^36^ Our finding was supported this previous conclusion that CTLs steadily increasing trend with the lowest number present in normal tissue, higher numbers in successive stages, especially the highest in HSIL significantly. Activated memory CD4^+^ T cells indicate favorable outcomes, whereas the adverse outcomes are highly associated with resting memory CD4^+^ T cells.^37^ A remarkable decrease happened in NK cells from HSIL to SCC in our study, which was reasonable with the shrinkage of the activated receptor (NKG2D) population from SCC to healthy donors.^38^ Of note, activated NK cells and activated mast cells suggest adverse outcomes in cervical cancer.^37^ Meanwhile, the contradictory changing trend of neutrophils in our study remaining further explored by enlarging the sample numbers. Further, we found MHC class II genes associated with upregulated function from normalcy to HSIL supported by a previous study,^39^ and interferon‐γ response was updated from normalcy to LSIL and HSIL. It is widely acknowledged that the innate cells recognized foreign antigens by kinds of receptors, including the Toll‐like receptors (TLRs). Activated TLRs on APCs can trigger downstream signaling events leading to the expression of inflammatory cytokines and chemokines encompassing the interferon‐γ.^40^ This production of inflammatory cytokines (e.g., IFN‐γ secreted by NK cells or Th1 cells) contributes to upregulate the expression of the MHC II complex on CD4^+^ T cells, annotating the malignant transformation from immunogenic to tolerogenic in the HSIL stage.^39^ To sum up, the cytotoxic immune subpopulation exists decreasing abundance along with cervical tumorigenesis, and HSIL is the turning point during cervical carcinogenesis.

Furthermore, the local adaptive immune‐suppression should be noted.^29^ Previous studies showed the abundance of Tregs^5^, ^10^, ^41^ and M2 shows a gradual increase in cancerous development.^42^ Intriguingly, B lineage cells increasingly infiltrated in both HSIL and SCC stages,^43^ despite the dysfunction of humoral responses, were discovered in SCC. Moreover, a shift from a Th1 to a Th2 cytokine response was observed in HSIL and cancer compared to normalcy and LSILs relatively,^44^ leading to suppressive mechanisms such as the immaturation of DC cells, differentiation of CD4^+^ T cells to Tregs and polarization of immunosuppressive macrophage.^45^ Tregs residing in cervical precursor lesions inversely correlate with spontaneous regression of preinvasive lesions regardless of the HPV subtype^46^ and M2‐macrophages correlated positively with unfavorable clinical outcomes.^47^ All that mentioned is instrumental in the predominance of immunosuppressive cells in both HSIL and SCC stages.

Even though tumor‐infiltrating lymphocytes express costimulatory markers, immunosuppressive checkpoints, which exhausted T cells and minimized responses to immune checkpoint inhibitors, are also expressed in cervical cancer. Recent studies show *PD*‐*1* expression on cervical T cells was associated and increased in parallel with increasing preinvasive lesion grade, aiding in the developing strategies based on the immune‐checkpoint pathway for immunotherapy of cervical disease.^44^, ^48^ Furthermore, *PD*‐*1*, *TIM*‐*3*, *CD28*, and *CD40* proteins were positively associated with genital inflammation.^49^ Our study demonstrated there were various inhibitory interleukins expression values strongly associated with immune infiltrating status in HSIL and SCC, indicating immune escape occurred before tumor invasion and revealing the relevant immune biomarkers for treatment.

Recently, the critical clinical benefit of checkpoint immunotherapy has been obtained in various treatment plans.^50^ Over the past 5 years, the clinical success of ICIs was tested in malignant diseases such as melanoma, nonsmall cell lung carcinoma and renal cell carcinoma.^51^ Meanwhile, a series of clinical trials explored the efficacy of single checkpoint inhibitors in cervical cancer.^52^ Ipilimumab, the CTLA‐4 inhibitor, characterized the safety and manageable toxicities in cervical cancers with below‐par performance, the same as nivolumab targeting PD‐1. Another PD‐1 inhibitor pembrolizumab had a consistent anti‐tumor activity and clinical safety and became the first approved checkpoint inhibitor for the treatment of cervical cancer.^35^ Here, identifying three distinct immune clusters can we dissect the immune pattern across the spectrum of cervical disease contributing to finding the most potential targetable checkpoints. Immune checkpoint therapy was recommended for both immune‐hot and immune‐warm but not immune‐cold clusters. The immune cold phenotype together with low immune infiltrates and low immune‐related gene expression may be the least responsive to immunotherapy, which resulted from defective immune priming and immunologic ignorance (lack of antigens and antigen presentation).^53^ Meanwhile, the immune‐warm group ranked the second of infiltrating lymphocytes score and checkpoints expression after the immune‐hot group accompanied with the highest infiltration of T cells. Moreover, the immune‐hot cluster with significant immune cell infiltrates and immune‐related gene expression susceptible to classic immune checkpoint blockade, concomitant with the relative paucity of T cells. Thus, we preferred to administrate the immune checkpoint blockade in immune‐hot and immune‐warm clusters based on tipping the balance between efficacy and toxicity.

Regarding the specific therapeutic targets, we firstly explored the immune‐hot and immune‐warm subtypes characterized by the elevated expression of immune checkpoints under the influence of both immunoreactive and immunosuppressed responses. Infiltrating pattern in immune‐hot phenotype was the less abundance of good‐prognostic cells but more adverse indicators, e.g., the increasing B lineage and decreasing CD8^+^ T cells and dysfunctional neutrophils. Furthermore, immune‐hot and immune‐warm clusters, also differed from the other modules, showed a suppressive milieu with overlappingly elevated expression of the top three immune checkpoints *TIGIT*, *CTLA4*, and *HAVCR2*. Meanwhile, costimulatory genes like *ICOS*, *CD48*, and *TNFSF13B* (also known as B‐cell activating factor, Figure 4F; Figure S4D) can be considered as potential targets. Inspection of our flow cytometry results, we found the percentage of PD‐1^+^ CD8^+^ T cells, CTLA‐4^+^ CD8^+^ T cells and IDO^+^ CD8^+^ T cells in CD3^+^ T cells grew significantly from normalcy to HSIL (Figure 4F); however, there was insignificant result in comparison of HSIL with normalcy of the percentage of TIM‐3^+^ CD8^+^ T cells and LAG‐3^+^ CD8^+^ T cells in CD3^+^ T cells (Figure 4F; Figure S4D). Together with the mentioned bioinformatic findings, it annotated that the anti‐IDO and anti‐CTLA4 may be regarded as potential therapeutic markers. When it came to the co‐stimulatory immune checkpoint molecule, *ICOS* could also be a candidate for its expression on activated T cells. Considering the downregulation of antimicrobial humoral response in cancer, we supposed the two B cell immuno‐stimulators, *CD48* and *TNFSF13B*, could be the potential targets as well. In general, immunotherapy in both immune‐hot and immune‐warm clusters was proved to be evidence‐based and promising.

There is room for improvement, as following parts: (i) analyses confined to the Affymetrix Human Genome U133 Plus 2.0 Array, and inclusion of independent platforms especially of RNA‐seq would improve the validity of the results; (ii) lack of association with survival or clinical parameters (e.g., stages for SCC) for cases with high immune cells infiltration or the immune‐hot, immune‐cold, or immune‐warm clusters; (iii) insufficient data contributed confounding factors; (iv) insufficient sample names and checkpoint markers like immune‐stimulators (such as ICOS) checked in our experiments.

To conclude, our study reveals a comprehensive immune landscape throughout the process from normalcy to squamous carcinoma cancer in the cervix. We proposed HSIL is the kick‐starter to ignite the immunosuppressive responses during carcinogenesis by activating immune checkpoints. Moreover, our findings provided novel ideas for improving the patients’ clinical response to therapy by identifying different immune phenotypes. Not only can we make immune therapy possible in the preinvasive stages but also promote personalized immunotherapy in the tumor.

## CONFLICT OF INTEREST

The authors declare no conflict of interest.

## Supporting information

Fig S1

Fig S2

Fig S3

Fig S4

Table S1

Table S2

Table S3

## Data Availability

Data are available in a public, open access repository. All data relevant to the study are included in the article or uploaded as [Link], [Link], [Link], [Link], [Link], [Link], [Link]. Cervical disease mRNA expression data from 225 cases with clinical data were downloaded from GEO (https://www.ncbi.nlm.nih.gov/geo/) in March 2020. All data generated that are relevant to the results presented in this article are included in this article.
