# Supplementary figures and images for: The immune landscape during the tumorigenesis of cervical cancer
Source: Cancer Med. 2021 Mar 10;10(7):2380–95. doi: 10.1002/cam4.3833 (PMC7982625; doi:10.1002/cam4.3833)

Figure S1

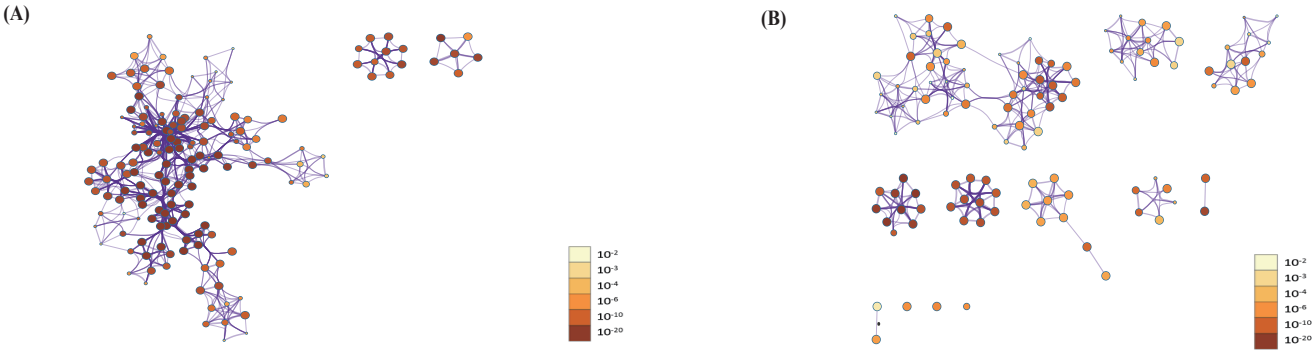

Supplement: Supplementary file 1 — Fig S1 [file CAM4-10-2380-s003.pdf]

**Figure S2**

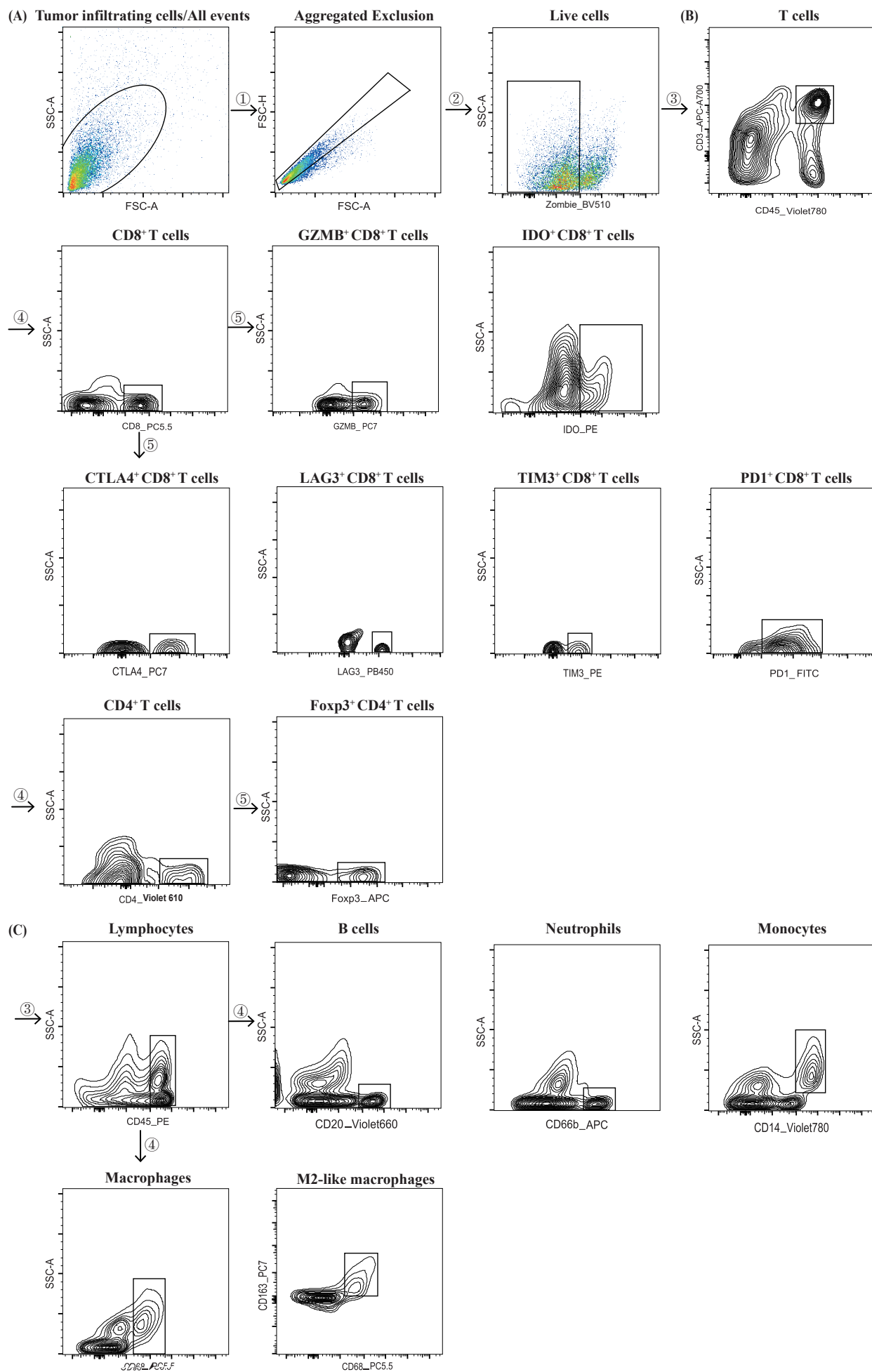

Supplement: Supplementary file 2 — Fig S2 [file CAM4-10-2380-s005.pdf]

Figure S3

(A) CIBERSORT LM22

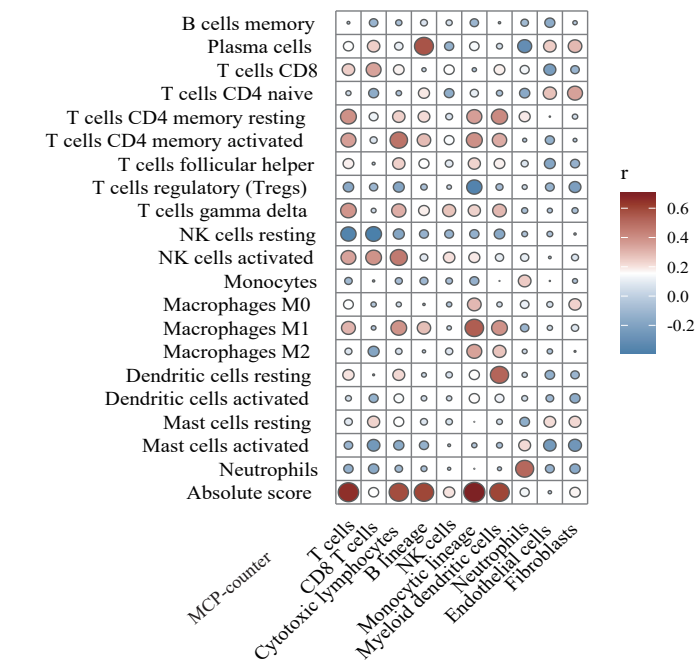

(B)

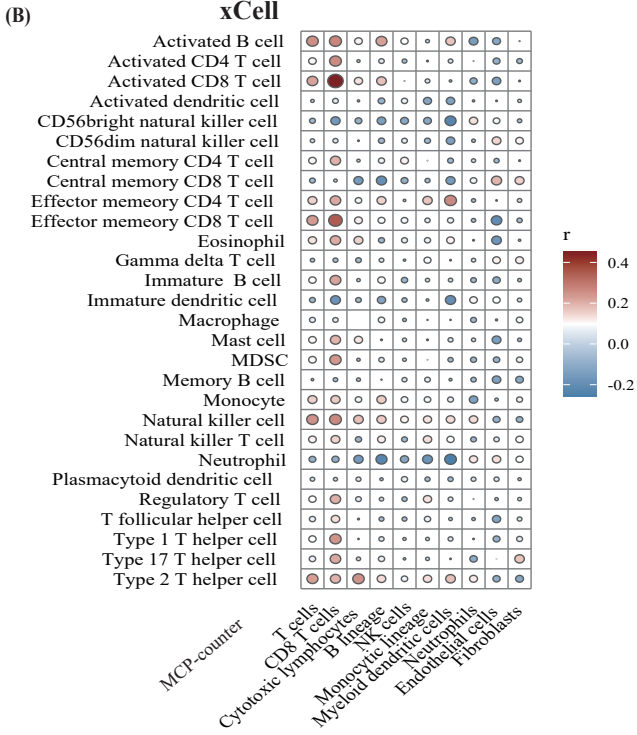

(C)

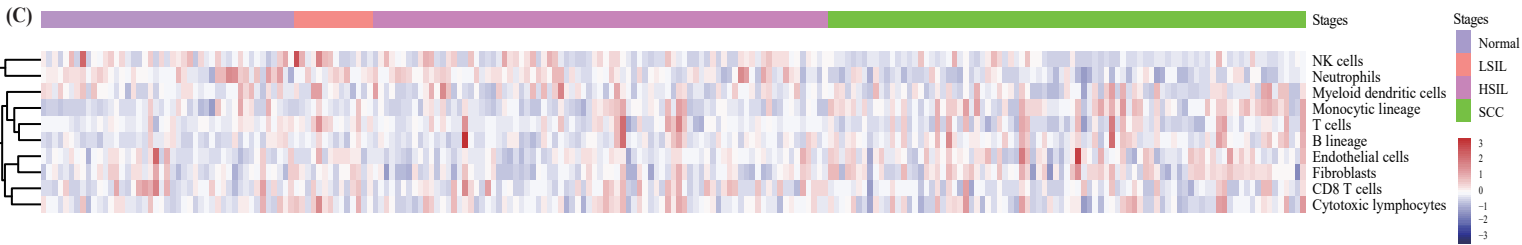

(D)

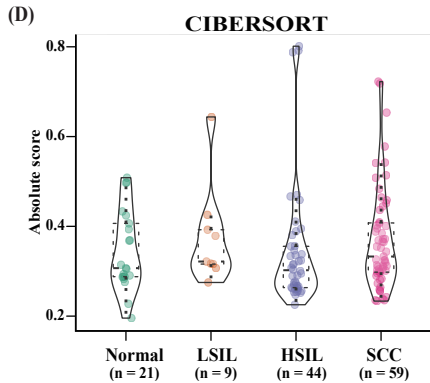

(E)

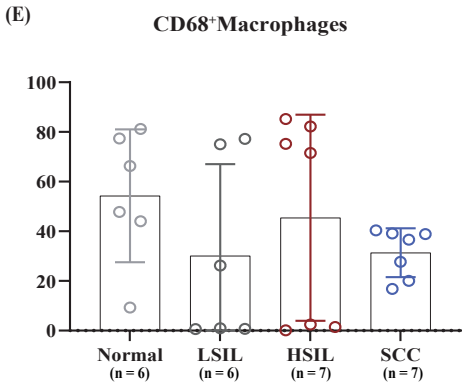

CD163+M2

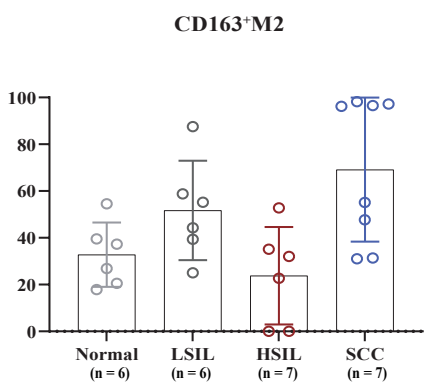

Supplement: Supplementary file 3 — Fig S3 [file CAM4-10-2380-s006.pdf]

**Figure S4**

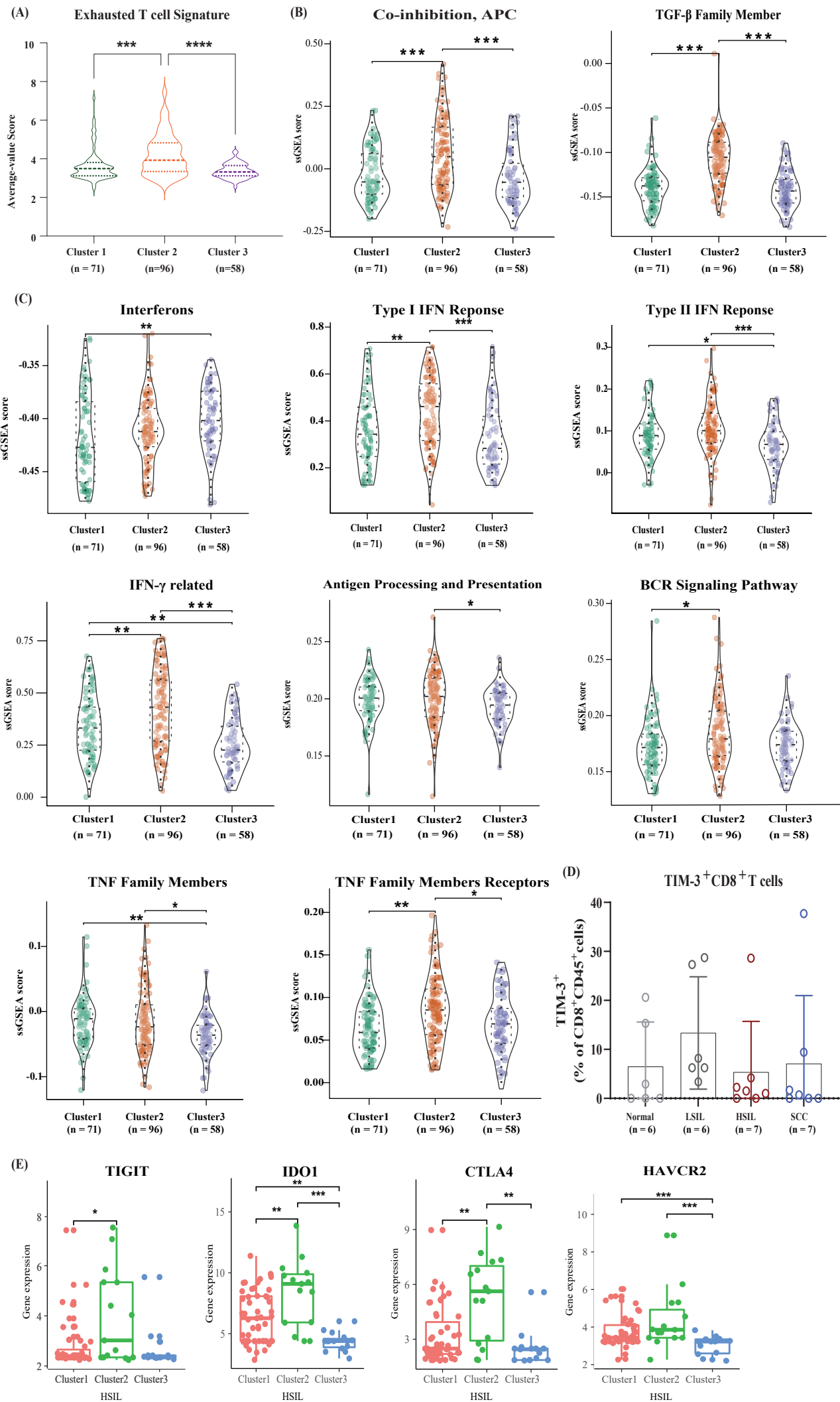

Supplement: Supplementary file 4 — Fig S4 [file CAM4-10-2380-s004.pdf]
