# Supplementary material for: The immune landscape during the tumorigenesis of cervical cancer
Source: Cancer Med. 2021 Mar 10;10(7):2380–95. doi: 10.1002/cam4.3833 (PMC7982625; doi:10.1002/cam4.3833)
Supplement: Supplementary file 5 — Table S1 [file CAM4-10-2380-s001.pdf]

Table S1. Clinical evaluation of 26 cervical specimens

	Normal (n=6, 23.0%)	LSIL (n=6, 23.0%)	HSIL (n=7, 27.0%)	SCC (n=7, 27.0%)
**Median age**	45	45.5	33.5	56
**HPV infection**				
HPV 16	1 (13.0)	1 (13.0)	3 (38.0)	3 (38.0)
HPV 18	0 (0.0)	2 (40.0)	1 (20.0)	2 (40.0)
other high-risk HPV	0 (0.0)	1 (25.0)	1 (25.0)	2 (50.0)
other HPV	1 (15.0)	1 (15.0)	3 (60.0)	0 (0.0)
uninfected	4 (66.7)	2 (33.3)	0 (0.0)	0 (0.0)

HSIL = high grade squamous intraepithelial lesion; LSIL = low grade squamous intraepithelial lesion; SCC= squamous cell carcinoma
