# Supplementary material for: The immune landscape during the tumorigenesis of cervical cancer
Source: Cancer Med. 2021 Mar 10;10(7):2380–95. doi: 10.1002/cam4.3833 (PMC7982625; doi:10.1002/cam4.3833)
Supplement: Supplementary file 6 — Table S2 [file CAM4-10-2380-s002.pdf]

Table S2. Monoclonal antibodies used for flow cytometry

No.	Antibody name	Company	Product No.	Marker
1	Brilliant Violet 785™ anti-human CD45 Antibody	Biolegend	368528	CD45
2	PE anti-human CD45 Antibody	Biolegend	304008	CD45
3	Alexa Fluor 700 anti-human CD3 Antibody antibody	Biolegend	300324	CD3
4	Brilliant Violet 605 anti-human CD4 Antibody	Biolegend	317438	CD4
5	APC anti-human CD4 Antibody	Biolegend	317416	CD4
6	PerCP/Cyanine5.5 anti-human CD8 Antibody	Biolegend	344710	CD8
7	PE/Cyanine7 anti-human/mouse Granzyme B Recombi	Biolegend	372214	GZMB
8	FOXP3 Antibody, APC	eBioscience	17-4777-42	Foxp3
9	FITC anti-human CD279 (PD-1) Antibody	Biolegend	329904	PD-1
10	PE anti-human CD366 (Tim-3) Antibody	Biolegend	345006	TIM-3
11	PE anti-mouse CD223 (LAG-3) Antibody	Biolegend	125208	LAG-3
12	PE/Cyanine7 anti-human CD152	Biolegend	369613	CTLA-4
13	IDO Antibody, PE	eBioscience	12-9477-42	IDO
14	FITC anti-human CD68 Antibody	Biolegend	333806	CD68
15	PE/Cyanine7 anti-human CD163 Antibody	Biolegend	333614	CD163
16	APC anti-human CD66b Antibody	Biolegend	305118	CD66b
17	Brilliant Violet 650 anti-human CD20 Antibody	Biolegend	302336	CD20
18	Brilliant Violet 785 anti-human CD14 Antibody	Biolegend	301839	CD14
