# Supplementary material for: The immune landscape during the tumorigenesis of cervical cancer
Source: Cancer Med. 2021 Mar 10;10(7):2380–95. doi: 10.1002/cam4.3833 (PMC7982625; doi:10.1002/cam4.3833)
Supplement: Supplementary file 7 — Table S3 [file CAM4-10-2380-s007.pdf]

Table S3: Basic information of datasets included in this study for charactering immune traits in progression of cervicla

Accession number /Source	Platform	Number of patients	Number of samples	Histology
GEO: GSE5787	Affymetrix Human Genome U133 Plus 2.0 Array	9	29	SCC:29
GEO: GSE63514	Affymetrix Human Genome U133 Plus 2.0 Array	128	128	Normal:24;LSIL:14;HSIL:62;SCC:28
GEO: GSE75132	Affymetrix Human Genome U133 Plus 2.0 Array	40	40	Normal:19;HSIL:21
GEO: GSE27678	Affymetrix Human Genome U133 Plus 2.0 Array	28	28	SCC:28
